# Incorporating evolutionary insights to improve ecotoxicology for freshwater species

**DOI:** 10.1111/eva.12507

**Published:** 2017-11-10

**Authors:** Steven P. Brady, Jonathan L. Richardson, Bethany K. Kunz

**Affiliations:** ^1^ Biology Department Southern Connecticut State University New Haven CT USA; ^2^ School of Forestry and Environmental Studies Yale University New Haven CT USA; ^3^ Department of Biology Providence College Providence RI USA; ^4^ U.S. Geological Survey Columbia Environmental Research Center Columbia MO USA

**Keywords:** acute exposure, adaptation, chloride, LC50, maladaptation, road salt runoff

## Abstract

Ecotoxicological studies have provided extensive insights into the lethal and sublethal effects of environmental contaminants. These insights are critical for environmental regulatory frameworks, which rely on knowledge of toxicity for developing policies to manage contaminants. While varied approaches have been applied to ecotoxicological questions, perspectives related to the evolutionary history of focal species or populations have received little consideration. Here, we evaluate chloride toxicity from the perspectives of both macroevolution and contemporary evolution. First, by mapping chloride toxicity values derived from the literature onto a phylogeny of macroinvertebrates, fish, and amphibians, we tested whether macroevolutionary relationships across species and taxa are predictive of chloride tolerance. Next, we conducted chloride exposure tests for two amphibian species to assess whether potential contemporary evolutionary change associated with environmental chloride contamination influences chloride tolerance across local populations. We show that explicitly evaluating both macroevolution and contemporary evolution can provide important and even qualitatively different insights from those obtained via traditional ecotoxicological studies. While macroevolutionary perspectives can help forecast toxicological end points for species with untested sensitivities, contemporary evolutionary perspectives demonstrate the need to consider the environmental context of exposed populations when measuring toxicity. Accounting for divergence among populations of interest can provide more accurate and relevant information related to the sensitivity of populations that may be evolving in response to selection from contaminant exposure. Our data show that approaches accounting for and specifically examining variation among natural populations should become standard practice in ecotoxicology.

## 1 INTRODUCTION

Ecotoxicology has a rich history of clarifying how organisms respond to environmental contaminants, with insights gleaned across developmental stages for a diverse suite of species (Walker, Sibly, Hopkin, & Peakall, 2012). Multidisciplinary perspectives and techniques, for example from genetics, physiology, and ecology, have yielded a robust understanding of toxicological outcomes and mechanisms across levels of biological organization, from molecular processes to population‐level and community‐level consequences (Peterson et al., 2003; Sturla et al., 2014; Whitehead, Pilcher, Champlin, & Nacci, 2012). Indeed, efforts aimed at understanding how molecular impacts of contaminants shape ecological outcomes have become a recent focus of ecotoxicology (e.g., “adverse outcome pathways,” Ankley et al., 2010).

In addition to contributing to a better understanding of contaminant effects in general, ecotoxicology has generated a large body of data that can be used to compare the sensitivity of different species to particular contaminants. Most of these comparisons are made possible through short‐term (i.e., “acute”) toxicity tests conducted under highly standardized conditions designed to estimate median lethal concentrations (LC50s) (Rand, Wells, & McCarty, 1995). These tests comprise an experimental exposure of organisms to a series of dilutions of a chemical to estimate the concentration causing 50% mortality. Typically, exposures are conducted for a period of 96 hr or less. In addition to being the most commonly used estimate of contaminant toxicity, LC50 values are routinely utilized in the development of policies regulating the allowable level of contaminants in the environment. For example, the United States Environmental Protection Agency (U.S. EPA) uses data from aquatic toxicity testing to develop National Recommended Water Quality Criteria (Firestone, Kavlock, & Zenick, 2009).

One aspect of biology that has seen relatively limited use in ecotoxicology is the influence of evolution on organismal sensitivity to toxic substances (Bickham & Smolen, 1994; Hammond, Jones, Stephens, & Relyea, 2012; Klerks, Xie, & Levinton, 2011), despite some early interest (e.g., Antonovics, Bradshaw, & Turner, 1971). This is surprising from the perspectives of both macroevolution and contemporary evolution. From a macroevolutionary perspective, the role for toxic selection pressures shaping adaptations and species evolution is thought to be substantial (Ogunseitan, 2005). Species diversification associated with natural selection imposed by toxic environments has been described for toxicants such as those from geological events (Peters & Gaines, 2012), as well as in the context of coevolutionary dynamics of plant and animal defenses (Futuyma & Agrawal, 2009). Thus, considering the substantial variation in contaminant sensitivities observed across extant groups of species (e.g., Kerby, Richards‐Hrdlicka, Storfer, & Skelly, 2010), we might expect that evolutionary relationships explain a portion of this variation and that more closely related species are more likely to share ecotoxicological trait phenotypes. This may be true regardless of whether contemporary contaminants acted as part of historical selective regimes. For example, if particular physiological mechanisms of decontamination are common across classes of contaminants and are relatively conserved across related lineages, more closely related species would be expected to show similar tolerances to a given contaminant. This potential influence of evolutionary history may be particularly relevant for chloride tolerance, which has evolved many times and for many species in the contexts of transitions from marine to freshwater habitats (Lee & Bell, 1999; Schluter, 2000). Indeed, all animal taxa evolved from marine ancestors, many of which have evolved into freshwater species (Little, 1990). Thus, macroevolutionary relationships should be viewed as valuable hypotheses for explaining and predicting patterns of toxicity across species.

In complement to macroevolutionary perspectives, contemporary evolutionary perspectives can also contribute to ecotoxicological insights by improving realism of toxicity estimates. This improvement stems from the fact that contemporary evolutionary perspectives acknowledge the capacity for local populations to diverge rapidly (i.e., over several generations) in traits and fitness due to responses to local environmental conditions (Carroll, Hendry, Reznick, & Fox, 2007; Kawecki & Ebert, 2004; Richardson, Urban, Bolnick, & Skelly, 2014). This fine‐scale variation across populations bears striking relevance for ecotoxicology: because environmental contaminants can be heterogeneously distributed in space, sensitivity to a given contaminant can differ from one population to the next depending on exposure history. These differences can be mediated by both evolutionary and plastic processes such as genetic, epigenetic, and maternal/environmental inheritance. Thus, estimates of species’ sensitivities (and variation therein) may not be well described by traditional approaches that ignore these evolutionary contexts when measuring toxicity. Instead, contaminants should be viewed not only as drivers of immediate lethal and sublethal effects, but also as potential agents of natural selection and/or transgenerational plasticity (Cothran, Brown, & Relyea, 2013; Dieter, 1993; Hua et al., 2015; Tanaka & Tatsuta, 2013). And thus, populations should be viewed as dynamic entities whose sensitivities can evolve. Notably, evolutionary and plastic changes can be adaptive or maladaptive (Ghalambor, McKay, Carroll, & Reznick, 2007). Thus, evolutionary processes can result in populations with increased or decreased tolerance.

Here, we evaluate the utility of both macro‐ and contemporary evolutionary approaches in the context of chloride toxicity in freshwater animals. Chloride is a widespread environmental contaminant that is particularly prevalent in roadside habitats where salt is used to de‐ice roads and/or control dust (Kaushal et al., 2005; Trombulak & Frissell, 2000). These practices have salinized many freshwater habitats in roaded landscapes (Kaushal et al., 2005). As a result, chloride concentrations have been increasing in freshwater bodies and are predicted to cause many Northeast and Midwest North American lakes to exceed the US EPA criterion for chronic chloride exposure (250 mg/L) by the year 2050 (Dugan et al., 2017). In addition to lethal effects, exposure of freshwater animals to elevated chloride concentrations in the 100s or 1,000s of mg/L can induce of a suite of sublethal effects. These effects include osmoregulatory disruption (Karraker & Gibbs, 2011a), diminished growth and development (Johnson et al., 2015), increased malformations and disease susceptibility (Brady, 2013; Hopkins, French, & Brodie, 2013; Karraker & Ruthig, 2009), and behavioral changes (Hall et al., 2017), all of which can influence fitness, community dynamics, and ecosystem function (Schuler et al., 2017; Van Meter & Swan, 2014).

Here, we use a phylogenetic approach to investigate whether chloride toxicity varies across species in a manner predicted by evolutionary relationships. Specifically, we compiled LC50 estimates of chloride toxicity from acute exposure studies for a suite of species of macroinvertebrates, amphibians, and fishes. We assessed whether phylogenetic signal of chloride toxicity is present among species. Next, we analyzed two acute exposure experiments designed to investigate population‐level variation in chloride toxicity (i.e., chloride LC50) for two amphibian species influenced by road salt runoff. We investigated whether road‐adjacent populations of *Ambystoma maculatum* (spotted salamander, Shaw) and *Rana sylvatica* (wood frog, *Rana sylvatica* = *Lithobates sylvaticus,* LeConte) have each evolved different chloride sensitivities compared to populations located away from roads. By comparing chloride sensitivity of multiple taxonomic groups and multiple populations of the same species, our approach allows us to address the utility of incorporating evolutionary perspectives into ecotoxicology.

## 2 MATERIALS AND METHODS

### 2.1 Macroevolution: Phylogenetic dataset and analysis of chloride toxicity

We compiled data on chloride LC50s for freshwater organisms using two primary sources—the Interspecies Correlation Estimate (ICE) Aquatic Toxicity Database v. 3.3 (Willming, Lilavois, Barron, & Raimondo, 2016) and the Canadian Water Quality Guidance for the Protection of Aquatic Life (Canadian Council of Ministers of the Environment 2011). The ICE Aquatic Toxicity Database is a collection of acute toxicity records maintained by the US EPA Gulf Ecology Division used to develop ecotoxicological models and predict toxicity of contaminants for species of interest. Studies listed in the primary database have passed a checklist of standardization criteria including requirements for chemical purity, test species, life stage, and exposure duration (Raimondo, Lilavois, Willming, & Barron, 2016). We examined all entries in the database for which the toxicant listed was “sodium chloride.” Sodium chloride entries in this database originated from a combination of peer‐reviewed literature, the US EPA 1988 Ambient Water Quality Criteria (AWQC) document for chloride (Benoit, 1988), and the ECOTOXicology database (U.S. Environmental Protection Agency 2016). Within the Canadian Water Quality Guidance document (Canadian Council of Ministers of the Environment 2011), we used sodium chloride LC50 values reported from studies that were used in the development of the short‐term exposure guideline for freshwater organisms. When LC50 values for a given species were reported as an average across multiple studies or tests, we examined the original references to identify individual LC50 estimates.

To supplement records from these databases, we searched Web of Knowledge using the phrase “(chloride and acute toxicity and freshwater and vertebrate* or invertebrate*).” We refined the search by the research area “environmental sciences ecology” and excluded document types “news” or “patent” or “meeting” or “abstract”. We screened results for studies that reported experimental exposures of freshwater aquatic organisms to sodium chloride and that also met inclusion criteria based on the ICE database criteria (Table S1). Finally, we also included unpublished estimates from acute sodium chloride studies (B. Kunz, unpublished data). When a given study provided multiple estimates for a single species, the mean value was used. Across this dataset, a linear model showed no difference in LC50 between 48‐, 72‐, and 96‐hr exposure periods (*F*_3,53_ = 0.641, *p *=* *.592). We therefore included estimates generated from any of these durations. This resulted in a total of 56 species including macroinvertebrates, amphibians, and fish (Table S2).

All analyses were conducted in R v. 3.1.1 (R Core Team, 2017). We used the R package “rotl” v. 3.0.3 (Michonneau, Brown, & Winter, 2016) to query and generate a subtree phylogeny from the current synthetic tree available in the Open Tree of Life (Hinchliff et al., 2015). We obtained a phylogenetic tree comprising 55 of the 56 species in our chloride toxicity dataset. Branch lengths were computed using the “Grafen” method (Grafen, 1989) implemented in the function “compute.brlen” in the R package “APE” v. 3.5 (Paradis, Claude, & Strimmer, 2004). The resulting tree was paired with species level chloride LC50s to investigate phylogenetic signal of chloride toxicity. Phylogenetic signal describes a pattern where the value of a given trait is more similarly distributed among closely related taxa than predicted by chance (Blomberg, Garland, & Ives, 2003). In other words, a phylogenetic signal would indicate that closely related species are similar in their sensitivity to chloride. We used the package “phytools” v. 0.6 (Zhang, Pei, & Mi, 2012) to evaluate the potential phylogenetic signal of chloride toxicity. Signal was inferred using both Pagel's lambda, a parameter that scales the correlation between species (Pagel, 1999), and Blomberg's K, a metric consisting of the ratio of phylogenetic dependence explained by a given tree to that expected by a null model of trait evolution (Blomberg et al., 2003). A random walk Brownian motion process is used as the null evolutionary model for both metrics. Because similar traits can arise independently across lineages, we conducted this analysis at different taxonomic levels. First, we evaluated phylogenetic signal across the main tree (i.e., including all 55 species). Next, using pruned trees, we evaluated phylogenetic signal within (1) the full suite of macroinvertebrates (*N* = 31), (2) each of three represented phyla (i.e., Chordata [*N* = 24], Arthropoda [*N* = 19], Mollusca [*N* = 9]), and (3) each class of chordates (i.e., fish [*N* = 11] and amphibians [*N* = 13]).

We further investigated the relationship between chloride toxicity and evolutionary distance between species using a Mantel matrix correlation test (Mantel, 1967). Mantel tests evaluate the correlation between pairwise values while accounting for nonindependence of cells by calculating significance by permuting the matrix columns and rows to obtain a simulated *p*‐value. Pairwise (between species) values of phylogenetic distance based on branch length were calculated using the function “cophenetic.phylo” in APE. Corresponding pairwise differences in chloride toxicity values were computed using the “dist” function in the R base package.

### 2.2 Contemporary evolution: Acute toxicity assays in natural populations

#### 2.2.1 Natural history and site selection

In complement to the synthetic phylogenetic analyses described above, we conducted our own sets of acute exposure assays to estimate chloride LC50 for 10 populations of *R. sylvatica* and *A. maculatum*. Results for *R. sylvatica* but not *A. maculatum* were previously reported by (Brady 2013) and are included here to highlight the potential generality in population‐level differences in sensitivity to an anthropogenic contaminant. Here, we describe natural history for both of these species, but focus on describing experimental methods for *A. maculatum*. Experimental methods for *R. sylvatica* closely mirror the methods used here for *A. maculatum* (see Brady, 2013).

Both *A. maculatum* and *R. sylvatica* are widely distributed throughout eastern North America, ranging from Quebec into the southeastern United States. Additionally, the range of *R. sylvatica* extends throughout much of Canada and Alaska and reaches into the Arctic Circle. Within our study region of northeastern Connecticut (see Brady, 2012, 2013), both species breed in late March or early April, when adults migrate from upland terrestrial habitat into ephemeral wetlands to reproduce. Whereas *R. sylvatica* in this region lay an average of approximately 800 eggs per egg mass (Brady, 2013; Richardson, 2012), *A. maculatum* lay ~200 eggs per female (Urban, 2010). *R. sylvatica* embryos develop over 2–3 weeks before hatching and continue to develop as aquatic larvae throughout spring and early summer until they metamorphose into terrestrial juveniles. *A. maculatum* embryos develop over 8–10 weeks before hatching and continue to develop as aquatic larvae throughout the summer, metamorphosing into terrestrial juveniles a month or more after that of *R. sylvatica*, typically in August or early September.

To obtain study organisms for acute exposure, we selected a suite of ten ponds—five roadside and five woodland. Roadside ponds were located <10 m from a paved road and were estimated to have a ~50‐year history of road salt‐based de‐icing practices (Brady, 2012; Transportation Research Board 1991). Woodland ponds were located >200 m from a paved road, away from the influence of road salt (Fig. S1). We selected roadside ponds with the highest specific conductance values in the region and containing breeding populations of each species. Specific conductance is a close proxy for road salt, and in this region averages around 1,100 μS in roadside ponds versus 30 μS in woodland ponds. Corresponding chloride concentration in roadside ponds during embryonic and larval development averaged 300 mg/L (range: 133–902 mg/L) compared to 3.31 mg/L (range: 3.08–3.49 mg/L) in woodland ponds. Woodland ponds were selected to complement roadside ponds in a pairwise fashion, maximizing similarity in abiotic factors and location. Four roadside‐woodland pond pairs were common to both *A. maculatum* and *R. sylvatica*, with one pair being unique for each species. A full description of site selection for these ponds is available in Brady (2012, 2013). Across all ponds, pairwise distance ranged from 880 to 6,060 m. Estimates of neutral genetic neighborhood for the wood frog range from about 1,000 to 10,000 m (Berven & Grudzien, 1990; Newman & Squire, 2001), although evidence for local adaptation has been reported between populations separated by as few as 10s of meters (Skelly, 2004). Thus, the populations we report likely experience limited gene flow.

#### 2.2.2 Experimental design

In spring 2009, we monitored ponds daily for the arrival of new *A. maculatum* egg masses. From each pond, we collected a subset of *A. maculatum* egg masses less than 48 hr old, with a target of 10 egg masses per pond. We selected egg masses that were spatially distributed and conspicuously large and distinct so as to avoid sampling masses that might have originated from the same female (Urban, 2008). While roadside egg masses appeared somewhat more compact than woodland egg masses (Karraker & Gibbs, 2011a,b), there were no conspicuous differences in the embryos themselves between sites, and selection of embryos from within each egg mass was haphazard. Thus, any potential effect of the <48‐hr exposure did not bias the subset of embryos employed in the experiment. Each egg mass was collected whole into a plastic 710‐ml container. Approximately 30 embryos were dissected out of each egg mass for separate experiments reported elsewhere (Brady, 2012). The remainder of each egg mass was retained within collection containers; natal water was replaced with aged, conditioned tap water. Each container was placed within separate 150‐L plastic wading pools that served as incubators, located in an outdoor enclosure covered with 50% shade cloth. Containers remained sealed, but were occasionally opened to allow air exchange. At the start of hatching, embryos and hatchlings were placed into 150‐L wading pools filled with aged, conditioned tap water. Animals were grouped by origin such that each wading pool corresponded to a single natural pond and contained the individuals from that pond. We initiated the acute exposure assay when most embryos hatched [ca. Harrison developmental stage 41 (Harrison, 1969)]. We exposed *A. maculatum* larvae to a series of five concentrations of road salt: 0.0 (aged, conditioned tap water), 6.3, 8.4, 10.7, and 12 g/L road salt composed of 99% or better purity NaCl. Based on the typical composition of sodium chloride, we estimated these treatments to contain 0.0, 3,800, 5,100, 6,500, and 7,300 mg/L Cl and found a specific conductivity of 126, 4,570, 8,890, 13,370, and 17,800 μS, respectively. From each pond, ten hatchlings were randomly stocked into each treatment and replicated across four experimental units per treatment (10 ponds × 5 concentrations × 4 replicates = 200 experimental units). A 5.1‐L container (33 × 20 × 11 cm) filled with 4 L of treatment water comprised each experimental unit. After 96 hr of exposure, we evaluated larval survival by response to prodding. Unresponsive larvae were deemed dead.

#### 2.2.3 Data analysis

All analyses were conducted in R v. 3.1.1. For each species, we used a generalized linear mixed model to first assess whether mortality varied with respect to salt concentration and population type (i.e., roadside vs. woodland). We modeled mortality as a bivariate response of failures and successes (i.e., mortality and survival). We assigned chloride concentration and population type as fixed effects. We used the package “lme4” (Bates, Mächler, Bolker, & Walker, 2015) to evaluate a suite of models differing only in random effect structure. Standard AIC selection criteria were applied to select the model for inference. The selected model for each species included both pond of origin and an index term representing each container as random effects; the latter was used to address overdispersion that is common in binomial mortality data. Inference was conducted with MCMC sampling using the package “MCMCglmm” v. 2.22.1 (Hadfield, 2010). Errors were characterized using the multinomial family. The prior for the residuals was the uninformative inverse Wishart distribution, while for the random effects, parameter‐extended priors were used (Hadfield, 2014; ch. 8). Following a significant effect of concentration × population type interaction in each species’ mixed model, we used a standard approach to estimate LC50 separately for each set of roadside and woodland populations. Specifically, we composed a generalized linear model characterized by a binomial error distribution with a logit link to estimate LC50 separately for each population type for each species. We used the package “MASS” v. 7.3.45 (Venables & Ripley, 2013) to compute the LC50 value from each model.

## 3 RESULTS

### 3.1 Phylogenetic signal

**Figure 1:**
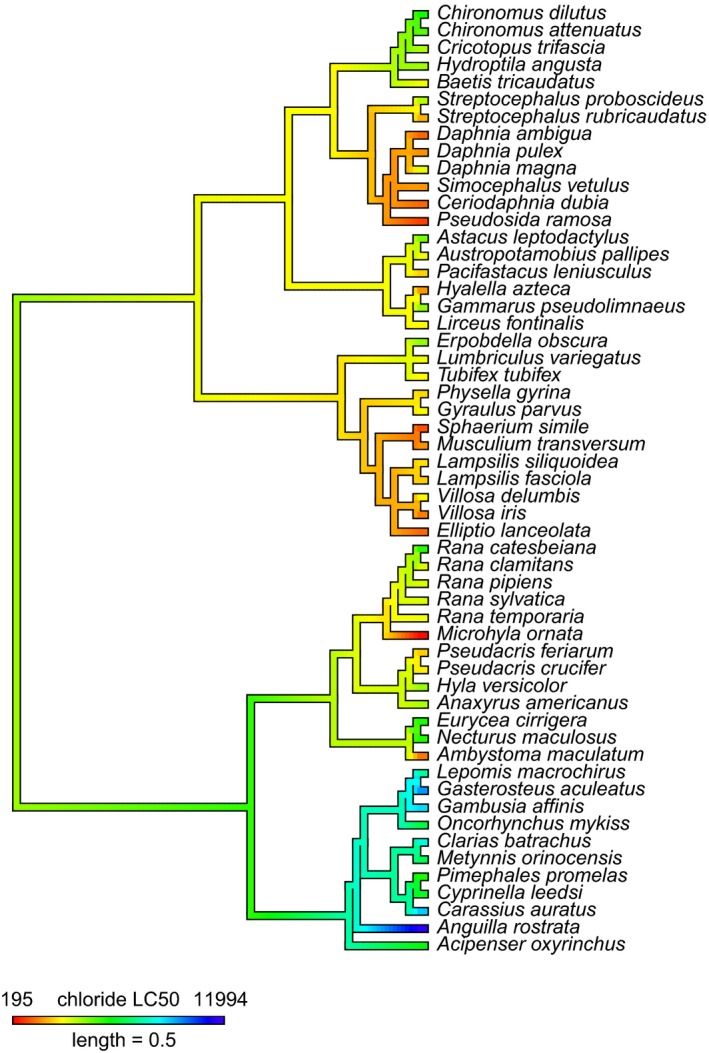
Chloride LC50 mapped to a phylogeny of freshwater organisms. A phylogenetic tree queried from the Open Tree of Life is shown. Mean chloride LC50 values (mg/L) for each species are mapped to the tree. LC50 values are color coded, with values indicated in the legend

We found evidence for a phylogenetic signal of chloride LC50 across the full tree comprising the complete complement of 55 species (Pagel's λ = 0.69, *p *<* *.001; *K *=* *0.23, *p *=* *.001; Figure 1). Similarly, Mantel tests indicated a significant association between phylogenetic relatedness and chloride toxicity similarity (*p *=* *.001). Pagel's λ is a parameter scaling the trait correlation between species relative to a correlation expected under the null model of Brownian evolution (e.g., a random walk process; Cavalli‐Sforza & Edwards, 1967; Felsenstein, 1985). λ values scale between 0 and 1, meaning that our λ of 0.69 indicates that evolution of chloride tolerance across species is explained, in part, by phylogenetic relationships. Blomberg's K is a parameter consisting of the ratio of phylogenetic dependence explained by a given tree to that expected under the same null model as Pagel's λ. Thus, a value near 1 indicates that related species resemble each other as much as expected by null evolution. Blomberg K values can be >1, signaling a similarity in traits among closely related species that is greater than expected by Brownian motion evolution. Values <1 indicate less similarity among closely related species than expected by the null model of evolution, as seen in our K of 0.23 for the full 55‐species tree (Blomberg et al., 2003; Garland, Bennett, & Rezende, 2005).

Importantly, the specific relationship between phylogeny and chloride tolerance differed among the taxonomic groups evaluated (Table S3). Chordates (i.e., fish and amphibians) in our dataset showed phylogenetic signal and distance‐based correlation consistent with the full tree (Pagel's λ = 0.63, *p *<* *.001; *K *=* *0.44, *p *=* *.001; Mantel *p *=* *.001). However, across the phylogeny containing only macroinvertebrates, variation in chloride toxicity was not explained by phylogenetic relationships (Pagel's λ = 0.50, *p *=* *.189; *K *=* *0.14, *p *=* *.146; Mantel *p *=* *.673). Similar results showing no evidence of phylogenetic signal were obtained for phyla‐level analyses of mollusks (Pagel's λ = 0.00, *p *=* *1.00; *K *=* *0.33, *p *=* *.438; Mantel *p *=* *.847) and arthropods (Pagel's λ = 0.39, *p *=* *.206; *K *=* *0.217, *p *=* *.326; Mantel *p *=* *.279).

### 3.2 Acute toxicity assays

**Figure 2:**
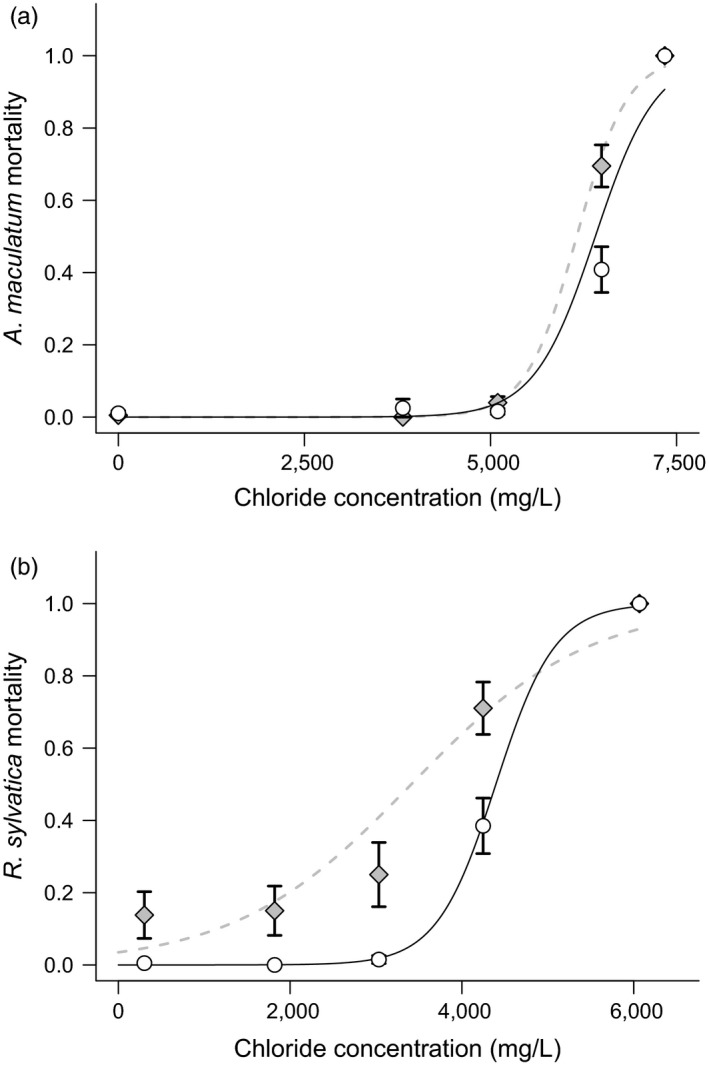
Larval amphibian mortality following acute exposure to road salt. The *y*‐axis shows the proportion of (a) *Ambystoma maculatum* and (b) *Rana sylvatica* larvae dead after 4 days of exposure to road salt. Each point (±1 SE) represents the mean among experimental units. Concentrations of total chloride (mg/L) are shown. Open circles represent woodland populations, while shaded diamonds represent roadside populations. Solid black lines (woodland) and dashed gray lines (roadside) represent fitted values from the generalized linear models

Mortality varied with respect to the interaction of population type and concentration for both *A. maculatum* (Posterior mean = 0.67, 95% CI = 0.12 to 1.24, *P*_*mcmc*_ = 0.01) and *R. sylvatica* (posterior mean = 0.62, 95% CI = −0.07 to 1.22, *P*_*mcmc*_ = 0.057). For both species, overall mortality was higher for roadside compared to woodland populations (Figure 2). Thus, for these two species of pond‐breeding amphibians, animals originating from roadside environments have a lower tolerance to experimental road salt exposure than those animals originating from the woodland environment. Similarly, for both species, LC50s were lower for roadside versus woodland populations (Figure 2). Specifically, *A. maculatum* larvae originating from roadside populations had a chloride LC50 of 6,158 mg/L (95% CI = 6,062–6,254), whereas larvae from woodland populations had an LC50 of 6,408 mg/L (95% CI = 6,295–6,521. For *R. sylvatica*, chloride LC50 in roadside populations was 3412 (95% CI = 3,240–3,583) compared to 4,395 (95% CI = 4,296–4,495) in woodland populations.

## 4 DISCUSSION

We found evidence for phylogenetic signal of chloride toxicity across the 55 species included in our comparative analysis. We also found that populations of amphibians experiencing decades of environmental chloride contamination from road salt runoff differed in their sensitivities to chloride compared to nearby low‐salt populations located away from roads. Together, these results highlight how perspectives from contrasting scales of evolutionary change (i.e., macroevolution and contemporary evolution) can reveal important variation in toxicity for environmental contaminants. Whereas phylogenetic signal can help build predictive knowledge for species lacking LC50 estimates and potentially point toward shared mechanisms mediating responses, contemporary evolutionary approaches can help refine our estimates for populations within a species facing variable levels of environmental contamination.

Both measures of phylogenetic signal (Pagel's λ and Blomberg's K) indicated a correlation between variation in chloride tolerance and evolutionary relationship across the main phylogeny of 55 species (Figure 1). Generally, as Pagel's λ increases from 0 to 1, so too does the strength of the association between trait variation and phylogenetic relationships, as seen in our data. Similarly, increasing values of Blomberg's K indicate increasing phylogenetic signal (although K can exceed 1 when variation is distributed among rather than within clades). When trees were pruned to analyze relationships within phyla, only species nested within the chordate phylum had a significant relationship between chloride tolerance and phylogeny. These different signals at different phylogenetic scales suggest that most of the variation in chloride toxicity occurs within divergent clades. Figure 1 illustrates that tolerance is particularly different within chordates between amphibians and bony fish. When we isolate those same lineages, the relative variation in trait value across species may be reduced below what can be distinguished using phylogenetic‐trait association metrics like λ and K (Table S3). Notably, Pagel's λ is more sensitive than Blomberg's K in detecting phylogenetic signal with fewer species in a dataset and with more complex evolutionary scenarios (Münkemüller et al., 2012); however, our results using Blomberg's K are qualitatively the same.

The variability in association between evolutionary relationship and chloride toxicity across different groupings within our dataset is somewhat surprising because phylogenetic signal is expected to be common (Blomberg et al., 2003). In a synthesis of published trees, Blomberg et al. (2003) report that 92% of traits show phylogenetic signal and that most traits show less signal than expected by the null model of random Brownian motion evolution. Analytically, a lack of phylogenetic signal can arise from a lack of power, which increases steeply with phylogeny size. Blomberg et al. (2003) show that a power of 0.8 is typically reached with 17–20 species in a tree. Thus, power is likely not limiting our ability to detect a signal in the overall macroinvertebrate tree (*N* = 31 species). However, power may be insufficient in the phyla‐level macroinvertebrate trees, which contain fewer species. Several evolutionary processes can also result in trees with no phylogenetic signal. Convergent evolution to similar environmental variables is one process that can lead to distantly related species having similar trait distributions (Wake, 1991). For example, we see strong similarities in chloride tolerance between distantly related groups of freshwater mussels (e.g., *Villosa* spp.) and water fleas (e.g., *Daphnia* spp.). Character displacement can also result in dissimilarities between closely related species (Losos, 2000). Additionally, the relationships in our macroinvertebrate phylogeny span three phyla (Annelida, Arthropoda, and Mollusca). Resolving phylogenetic signal across this level of diversity will likely require toxicity estimates for additional species to span more proximate evolutionary relationships.

In light of the phylogenetic signal we detected across our primary tree, we suggest that phylogenetic analyses become more routinely employed in ecotoxicology (e.g. Carew, Miller, & Hoffmann, 2011). The results here present an initial assessment of the potential for phylogenetic analyses to predict species responses. The utility of this approach appears limited primarily by the relatively large evolutionary distance between diverse groups of macroinvertebrates species. Ideally, future toxicity studies will provide estimates of chloride toxicity for species within groups that are poorly represented or absent from this phylogeny (e.g., Palaeoptera and Diptera). This would require ecotoxicologists to be cognizant of the phylogenetic relationships among taxa with corresponding chloride toxicity and to specifically target those that are not represented. Notably, this approach of choosing study species for toxicity testing based on phylogenetic representation would differ from typical approaches, which tend to focus on model organisms and/or species of concern.

For both *R. sylvatica* and *A. maculatum*, chloride LC50s were lower among those populations originating from roadside ponds (Figure 2). Thus, the populations that are actually exposed to chloride contamination in their environment are more sensitive than populations from uncontaminated habitats. That these divergent outcomes occurred for two different species suggests that differentiation between populations may be common in the context of anthropogenic chloride contamination. Moreover, the fact that for one species, relative chloride sensitivity ran counter to evidence for local adaptation to road adjacency (Brady, 2012) suggests that LC50s may be incomplete predictors of toxic effects in the wild.

The reduced tolerance to chloride shown by roadside populations is surprising. We would expect road salt contamination in the environment to act as an agent of natural selection, resulting in elevated not diminished tolerance to chloride (Brady & Richardson, 2017). Indeed, survival of both *R. sylvatica* and *A. maculatum* larvae has been shown to decline with chronic exposure to chloride at concentrations ranging from 145 to 628 mg/L (Karraker, Gibbs, & Vonesh, 2008; Sanzo & Hecnar, 2006). These concentrations are well within the ranges found in the roadside ponds reported here (133–902 mg/L), suggesting that road salt is likely causing mortality in wild populations and could indeed be acting as an agent of natural selection. In light of the increased sensitivity among roadside populations, processes such as inherited environmental effects or transgenerational plasticity may be acting counter to selection, negatively influencing larval tolerance to chloride.

The increased sensitivity to road salt is particularly surprising for *A. macululatum*, which are locally adapted to road adjacency (Brady, 2012). In the context of a reciprocal transplant experiment conducted across the same populations as those reported here, roadside populations survive at higher rates in their natal ponds compared to transplanted woodland populations (Brady, 2012). Together, these contrasting outcomes between laboratory‐based acute exposures and field‐based reciprocal transplants suggest that local adaptation may result from selection pressures other than road salt, or that acute exposure assays may be too simplified relative to natural conditions to capture important influences on fitness associated with roadside dwelling (e.g., Chapman, 2000). Even if road salt is an important selection agent in roadside ponds, the context in which it acts may be critical in shaping selection and local adaptation. For example, whereas woodland populations better tolerate chloride in the laboratory, roadside populations may better tolerate chloride in the field, where multiple stressors such as predation and competition are at play (e.g., Relyea & Mills, 2001). Relatedly, the difference in mortality in the acute exposures reported here occurred at chloride concentrations above that found in roadside ponds and thus might not correlate with fitness effects in the wild. These context dependencies highlight the abstract nature of acute toxicity assays and suggest that such approaches may bear limited relevance to real‐world outcomes.

For *R. sylvatica*, the difference in LC50s between populations is less surprising. Contrasting the local adaptation pattern of *A. maculatum*,* R. sylvatica* populations used in this study show evidence of local maladaptation in reciprocal transplant and common garden experiments (Brady, 2013, 2017). Compared to woodland populations, roadside populations survive at lower rates in their natal ponds and accrue more developmental malformations when chronically exposed to road salt. These maladaptive responses (including decreased tolerance to chloride) could result from negative transgenerational effects, for example, if parental exposure to the roadside environment compromises offspring viability via contaminant transfer and/or epigenetic effects (Head, Dolinoy, & Basu, 2012; Metts, Buhlmann, Scott, Tuberville, & Hopkins, 2012). Alternatively, maladaptive responses could occur if roadside selection pressures are strong enough to reduce population size and result in inbreeding depression (Falk, Parent, Agashe, & Bolnick, 2012). Regardless of mechanism, reduced chloride tolerance suggests that exposure of prior generations to the roadside environment imparts negative effects on subsequent generations. Future research elucidating these mechanisms will benefit potential mitigation efforts. For example, whereas maternal effects might be alleviated by environmental restoration efforts that reduce chloride contamination, genetic‐based maladaptation might require additional efforts such as targeted gene flow between populations with similar natural selection environments.

Few studies of acute toxicity explicitly test for variation at the population level, yet our data show that variation among populations can be on par with that found between species. Similar intraspecific variation in response to contaminants is reported elsewhere in the literature. Bridges and Semlitsch (2001) show that tolerance to a common pesticide varies among populations of *R. sphencocephala* (*Rana sphencocephala*, southern leopard frog, Cope) more than among congeneric species of frogs. Specifically, across nine *Ranid* species, mean time to death following exposure to a single concentration of carbaryl ranged from 5 to 34 hr. Comparatively, mean time to death among ten populations of *R. sphencocephala* ranged from 6 to 45 hr. Still, other cases show that some populations are relatively uniform in their response to contaminants. For example, Hammond et al. (2012) show that tolerance to endosulfan—an insecticide that is highly toxic to amphibians (Relyea, 2009)—varies little among six populations of *R. sylvatica*. Notably however, these populations were not sampled to represent variation in endosulfan exposure histories.

Here, despite showing difference in chloride LC50s comparable to differences seen between species, the local populations we studied were separated by just hundreds of meters (Fig. S1). Such microgeographic differentiation in contaminant sensitivity has received very little attention, yet can have profound impacts on the relevance of toxicity assessments and end points. Despite the fact that the majority of our ecotoxicological insights are derived from the simplified contexts of laboratory‐based exposure experiments, results are often interpreted as generalizable to the species level. Moreover, because acute exposure experiments typically utilize cultured or locally collected study organisms with no history of exposure, responses may not well represent natural populations where evolution and/or transgenerational plasticity mediate outcomes. Although the magnitude of differences in LC50s between local populations described here would likely not drive numerically different water quality criterion for chloride following EPA guidelines (Stephan et al., 1985), it is important to note that these differences emerged after only ~50 years of road de‐icing. Thus, it is reasonable to speculate that over time, larger differences may evolve (or in the case of inherited environmental effects, may accumulate) particularly as chloride levels in surface waters continue to rise (Corsi, De Cicco, Lutz, & Hirsch, 2015).

## 5 CONCLUSION

Together, the analyses presented here demonstrate that variation in sensitivity to contaminants is widespread both across and within species. Explicit studies on evolution and evolutionary divergence provide a critical perspective that helps resolve this variation. Variation among species is explained in part by their phylogenetic relationships; still more variation can be explained by considering within‐species responses associated with local conditions and exposure history. These results support the premise that incorporating evolutionary perspectives in ecotoxicology studies will not only improve ecotoxicological insights, but indeed are critical to accurately predict responses to contaminants. Whereas phylogenetics can provide inference into relationships among species, contemporary evolutionary approaches can refine insight to more accurately account for how populations are responding dynamically to environmental contaminants. We hope that these powerful evolutionary approaches will be integrated with standard ecotoxicological methods to improve the accuracy of environmental contaminants assessments.

## DATA ARCHIVING STATEMENT

Data for this study are available from the Dryad Digital Repository: https://doi.org/10.5061/dryad.4k66s.
