# Supplementary material for: Incorporating evolutionary insights to improve ecotoxicology for freshwater species
Source: Evol Appl. 2017 Nov 10;10(8):829–38. doi: 10.1111/eva.12507 (PMC5680426; doi:10.1111/eva.12507)
Supplement: Supplementary file 1 [file EVA-10-829-s001.docx]

**Supplementary Information**

**Figure S1. Study region and reciprocal pair design.** Location of 12 total ponds from which populations were sourced for acute chloride exposure is shown on a map of the region. Red symbols indicate roadside ponds; blue symbols indicate woodland ponds. Each roadside-woodland pond pair shares a common symbol shape. Interstate highway (I-84) and on/off-ramp infrastructure is indicated in yellow. Primary roads are heavily shaded, while secondary roads are lightly shaded. Bar graph inset shows mean specific conductance (µS) (± 1 SE) for roadside (red) and woodland (blue) ponds. For roadside ponds, conductivity is shown as the average of surface and bottom values. This is because specific conductance in the bottom waters of roadside ponds is nearly twice that of surface water on average. No such vertical gradient exists in woodland ponds.

**
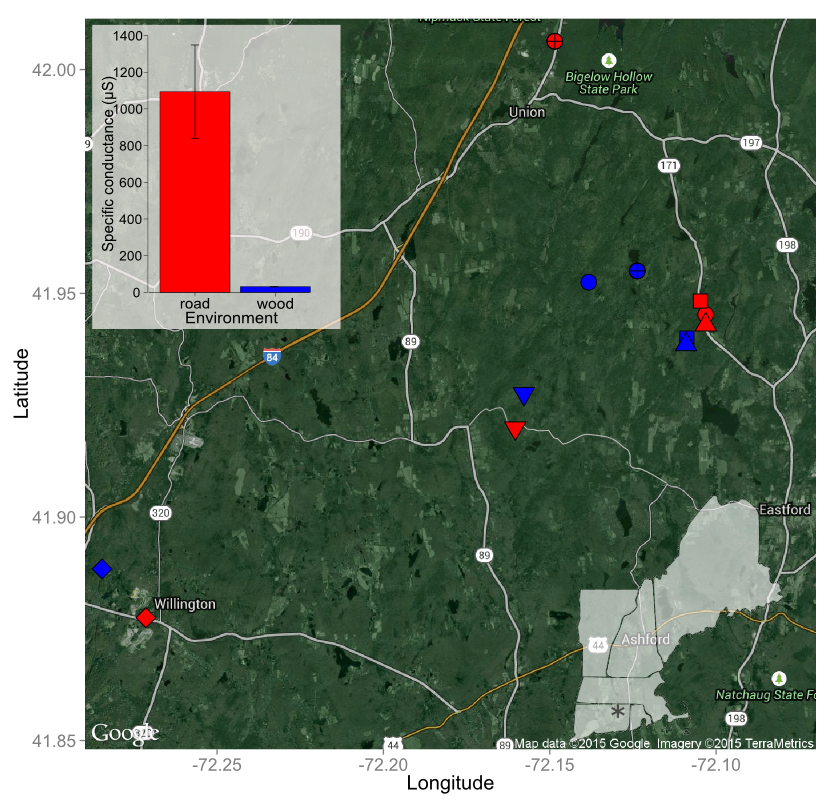
**

**Table S1.** Selection criteria for inclusion of acute toxicity values from the literature in the phylogenetic analysis (table modified from Raimondo et al. 2016). Criteria represent a subset of standardization criteria listed for inclusion in the primary ICE database. Requirements are identical, except that our dataset was limited to sodium chloride records and a more restrictive set of life stages.

| **Category** | **Data information** | **Criteria** |
| --- | --- | --- |
| Test chemical | Identity | CAS:7647-14-5 Chemical name: sodium chloride |
|  | Compound | Single compound tested; mixtures excluded |
|  | Purity | >90%, reagent/analytical grade or equivalent |
| Organism | Species | Fish, aquatic invertebrates, amphibians |
|  | Life stage | All species: eggs excluded Fish: primarily juveniles Invertebrates: juveniles and glochidia (mussels) Amphibians: aquatic larvae |
| Test conditions | Test media | Aquatic (no sediment, dietary, mixed dose or phototoxicity) |
|  | Exposure type | Static, flow-through, or static renewal |
|  | Exposure duration | Acute; 48 or 96 hours |
|  | Endpoint | EC50 or LC50 |
|  | Endpoint values | ~, > or < excluded |
|  | Measurement | Immobility or mortality |

**Table S2.**  Exposure duration (hours) and median effect concentrations (i.e. LC50s) used in phylogenetic analyses, sorted by taxa and species. All effect concentrations are reported in mg chloride per liter (mg Cl/L). The source reference is indicated for each LC50; full citation information for each reference is provided immediately after table.

| Taxa and species | | Test duration (hours) | LC50 or EC50 (mg Cl/L) | Study |
| --- | --- | --- | --- | --- |
| Amphibian | |  |  |  |
|  | *Ambystoma maculatum* | 96 | 1178 | Collins & Russell 2009 |
|  | *Bufo americanus* | 96 | 3926 | Collins & Russell 2009 |
|  | *Eurycea cirrigera* | 96 | 5657 | B. Kunz, personal communication 2016 |
|  | *Hyla versicolor* | 96 | 4312 | B. Kunz, personal communication 2016 |
|  | *Microhyla ornata* | 96 | 195 | Padhye & Ghate 1992 |
|  | *Necturus maculosus* | 96 | 5657 | B. Kunz, personal communication 2016 |
|  | *Pseudacris crucifer* | 96 | 2830 | Collins & Russell 2009 |
|  | *Pseudacris feriarum* | 96 | 2320 | Garibay & Hall 2004 |
|  | *Rana catesbeiana* | 96 | 5846 | ENVIRON International Corporation 2009 |
|  | *Rana clamitans* | 96 | 3109 | Collins & Russell 2009 |
|  | *Rana pipiens* | 96 | 3385 | Jackman 2010 |
|  | *Rana sylvatica* | 96 | 3617 | B. Kunz, personal communication 2016 |
|  | *Rana sylvatica* | 96 | 3887 | Brady 2013 |
|  | *Rana sylvatica* | 96 | 1721 | Collins & Russell 2009 |
|  | *Rana sylvatica* | 96 | 3617 | Harless et al. 2011 |
|  | *Rana sylvatica* | 96 | 3099 | Sanzo & Hecnar 2006 |
|  | *Rana temporaria* | 96 | 3140 | Viertel 1999 |
| Fish |  |  |  |  |
|  | *Acipenser oxyrinchus* | 96 | 5906 | King & Farrell 2002 |
|  | *Anguilla rostrata* | 96 | 11994 | Hinton & Eversole 1979 |
|  | *Carassius auratus* | 96 | 9456 | Threader & Houston 1983 |
|  | *Clarias batrachus* | 48 | 8518 | Komarudin et al. 1992 |
|  | *Cyprinella leedsi* | 96 | 6070 | ENVIRON International Corporation 2009 |
|  | *Gambusia affinis* | 96 | 9099 | Al-Daham & Bhatti 1977 |
|  | *Gasterosteus aculeatus* | 96 | 10200 | Garibay & Hall 2004 |
|  | *Lepomis macrochirus* | 96 | 5840 | Birge et al. 1985 |
|  | *Lepomis macrochirus* | 96 | 7898 | Patrick et al. 1968 |
|  | *Lepomis macrochirus* | 96 | 7847 | Trama 1954 |
|  | *Metynnis orinocensis* | 96 | 6461 | Velasco-Santamaria & Cruz-Casallas 2008 |
|  | *Oncorhynchus mykiss* | 96 | 6031 | Elphick et al. 2011 |
|  | *Oncorhynchus mykiss* | 96 | 6744 | R. Spehar memorandum to C. Stephan 1987 |
|  | *Pimephales promelas* | 96 | 6570 | Birge et al. 1985 |
|  | *Pimephales promelas* | 96 | 4080 | Elphick et al. 2011 |
|  | *Pimephales promelas* | 96 | 4371 | Meier & Blastos 2001 |
|  | *Pimephales promelas* | 96 | 6916 | Meyer et al. 1985 |
| Macroinvertebrate | |  |  |  |
|  | *Astacus leptodactylus* | 48 | 5497 | Firkins 1993 |
|  | *Astacus leptodactylus* | 72 | 4647 | Firkins 1993 |
|  | *Astacus leptodactylus* | 96 | 4320 | Firkins 1993 |
|  | *Austropotamobius pallipes* | 48 | 3525 | Firkins 1993 |
|  | *Austropotamobius pallipes* | 72 | 2754 | Firkins 1993 |
|  | *Austropotamobius pallipes* | 96 | 2572 | Firkins 1993 |
|  | *Baetis tricaudatus* | 48 | 3137 | Lowell et al. 1995 |
|  | *Ceriodaphnia dubia* | 48 | 1068 | Elphick et al. 2011 |
|  | *Ceriodaphnia dubia* | 48 | 965 | Harmon et al. 2003 |
|  | *Ceriodaphnia dubia* | 48 | 477 | Hoke et al. 1992 |
|  | *Ceriodaphnia dubia* | 96 | 1420 | Meier & Blastos 2001 |
|  | *Ceriodaphnia dubia* | 48 | 1189 | Mount et al. 1997 |
|  | *Ceriodaphnia dubia* | 48 | 1042 | Mount & Gulley 1992 |
|  | *Ceriodaphnia dubia* | 48 | 1351 | Soucek et al. 2011 |
|  | *Ceriodaphnia dubia* | 48 | 1288 | Warne & Schifko 1999 |
|  | *Chironomus attenuatus* | 48 | 4798 | Thornton & Sauer 1972 |
|  | *Chironomus dilutus* | 96 | 5868 | Elphick et al. 2011 |
|  | *Chironomus riparius* | 48 | 6912 | Wang & Ingersoll 2010 |
|  | *Cricotopus trifascia* | 48 | 3796 | Hamilton et al. 1975 |
|  | *Daphnia ambigua* | 48 | 1213 | Harmon et al. 2003 |
|  | *Daphnia magna* | 48 | 2565 | Beisinger & Christensen 1972 |
|  | *Daphnia magna* | 48 | 3165 | Davies & Hall 2007 |
|  | *Daphnia magna* | 48 | 2024 | Dowden 1961 |
|  | *Daphnia magna* | 48 | 3584 | Dowden & Bennett 1965 |
|  | *Daphnia magna* | 48 | 3631 | Elphick et al. 2011 |
|  | *Daphnia magna* | 48 | 2883 | Hoke et al. 1992 |
|  | *Daphnia magna* | 48 | 621 | Khangarot & Ray 1989 |
|  | *Daphnia magna* | 48 | 3325 | Martinez-Jeronimo & Martinez-Jeronimo 2007 |
|  | *Daphnia magna* | 48 | 3944 | Meyer et al. 1985 |
|  | *Daphnia magna* | 48 | 3422 | Mount et al. |
|  | *Daphnia magna* | 96 | 3877 | Mount et al. 1997 |
|  | *Daphnia pulex* | 48 | 1470 | Birge et al. 1985 |
|  | *Daphnia pulex* | 48 | 2042 | Gardner & Royer 2010 |
|  | *Elliptio lanceolata* | 96 | 1274 | Wang & Ingersoll 2010 |
|  | *Erpobdella obscura* | 96 | 4310 | ENVIRON International Corporation 2009 |
|  | *Gammarus pseudolimnaeus* | 96 | 4671 | Blasius & Merritt 2002 |
|  | *Gyraulus parvus* | 96 | 3043 | GLEC (Great Lakes Environmental Center) & INHS (Illinois Natural History Survey) 2008 |
|  | *Gyraulus parvus* | 96 | 3044 | Soucek et al. 2011 |
|  | *Hyalella azteca* | 96 | 1382 | Elphick et al. 2011 |
|  | *Hydroptila angusta* | 48 | 4040 | Hamilton et al. 1975 |
|  | *Lampsilis fasciola* | 96 | 2415 | Bringolf et al. 2007 |
|  | *Lampsilis siliquoidea* | 96 | 2767 | Bringolf et al. 2007 |
|  | *Lirceus fontinalis* | 96 | 2950 | Birge et al. 1985 |
|  | *Lumbriculus variegatus* | 96 | 3100 | Elphick et al. 2011 |
|  | *Musculium transversum* | 96 | 1930 | Soucek et al. 2011 |
|  | *Musculium transversum* | 96 | 1930 | US EPA 2010 |
|  | *Pacifastacus leniusculus* | 48 | 3458 | Firkins 1993 |
|  | *Pacifastacus leniusculus* | 72 | 2063 | Firkins 1993 |
|  | *Pacifastacus leniusculus* | 96 | 1723 | Firkins 1993 |
|  | *Physella gyrina* | 96 | 2540 | Birge et al. 1985 |
|  | *Pseudosida ramosa* | 48 | 838 | Freitas & Rocha 2011 |
|  | *Simocephalus vetulus* | 48 | 1867 | Loureiro et al. 2012 |
|  | *Sphaerium simile* | 96 | 902 | GLEC (Great Lakes Environmental Center) & INHS (Illinois Natural History Survey) 2008 |
|  | *Sphaerium simile* | 96 | 920 | Soucek et al. 2011 |
|  | *Streptocephalus proboscideus* | 48 | 4184 | Calleja et al. 1994 |
|  | *Streptocephalus rubricaudatus* | 48 | 1863 | Crisinel et al. 1994 |
|  | *Tubifex tubifex* | 96 | 5649 | Elphick et al. 2011 |
|  | *Tubifex tubifex* | 48 | 1567 | Khangarot 1991 |
|  | *Tubifex tubifex* | 96 | 1204 | Khangarot 1991 |
|  | *Tubifex tubifex* | 96 | 5144 | Soucek et al. 2011 |
|  | *Villosa delumbis* | 96 | 3173 | Bringolf et al. 2007 |
|  | *Villosa iris* | 48 | 1238 | Pandolfo et al. 2012 |
|  | *Villosa iris* | 96 | 1007 | Pandolfo et al. 2012 |
|  | *Villosa iris* | 96 | 1815 | Wang & Ingersoll 2010 |

*Full citations for Table S2*

Al‐Daham, N. K. and M. N. Bhatti. 1977. Salinity tolerance of Gambusia affinis (Baird & Girard) and Heteropneustes fossilis (Bloch). Journal of Fish Biology **11**:309-313.

Biesinger, K. E. and G. M. Christensen. 1972. Effects of various metals on survival, growth, reproduction, and metabolism of Daphnia magna. Journal of the Fisheries Board of Canada **29**:1691-1700.

Birge, W. J., J. A. Black, A. G. Westerman, T. M. Short, S. B. Taylor, D. M. Bruser, and E. D. Wallingford. 1985. Recommendations on numerical values for regulating iron and chloride concentrations for the purpose of protecting warmwater species of aquatic life in the Commonwealth of Kentucky. Memorandum of Agreement No. 5429. Kentucky Natural Resources and Environmental Protection Cabinet.

Blasius, B. J. and R. W. Merritt. 2002. Field and laboratory investigations on the effects of road salt (NaCl) on stream macroinvertebrate communities. *Environmental Pollution* 120 (2).

Brady, S. P. 2012. Road to evolution? Local adaptation to road adjacency in an amphibian (*Ambystoma maculatum*). Scientific Reports **2**.

Brady, S. P. 2013. Microgeographic maladaptive performance and deme depression in a fragmented landscape. PeerJ **1:e163**.

Bringolf, R. B., W. G. Cope, S. Mosher, M. C. Barnhart, and D. Shea. 2007. Acute and chronic toxicity of glyphosate compounds to glochidia and juveniles of *Lampsilis siliquoidea* (Unionidae). Environmental Toxicology and Chemistry **26**:2094-2100.

Calleja, MC, Guido Persoone, and P Geladi. 1994. Comparative acute toxicity of the first 50 multicentre evaluation of in vitro cytotoxicity chemicals to aquatic non-vertebrates. *Archives of Environmental Contamination and Toxicology* 26 (1):69-78.

Crisinei, A., L. Delaunay, D. Rossel, J. Tarradellas, H. Meyer, H. Saïah, P. Vogel, C. Delisle, and C. Blaise. 1994. Cyst-based ecotoxicological tests using Anostracans: Comparison of two species of Streptocephalus. *Environmental Toxicology and Water Quality* 9 (4):317-326.

Collins, S. J. and R. W. Russell. 2009. Toxicity of road salt to Nova Scotia amphibians. Environmental Pollution **157**.

Davies, T. D. and K. J. Hall. 2007. Importance of calcium in modifying the acute toxicity of sodium sulphate to *Hyalella azteca* and *Daphnia magna*. Environmental Toxicology and Chemistry **26**:1243-1247.

Dowden, B. 1961. Cumulative toxicities of some inorganic salts to Daphnia magna as determined by median tolerance limits. Pages 77-85 *in* Proceedings of the Louisiana Academy of Sciences.

Dowden, B. F. and H. J. Bennett. 1965. Toxicity of selected chemicals to certain animals. Journal (Water Pollution Control Federation) **37**:1308-1316.

Elphick, J. R., K. D. Bergh, and H. C. Bailey. 2011. Chronic toxicity of chloride to freshwater species: effects of hardness and implications for water quality guidelines. Environmental Toxicology and Chemistry **30**:239-246.

ENVIRON International Corporation. 2009. Chloride toxicity test results. Prepared for: Iowa Water Pollution Control Association. Project Number: #20-22235A. .

Firkins, I. 1993. Environmental Tolerances of Three Species of Freshwater Crayfish. University of Nottingham.

Freitas, Emanuela Cristina and Odete Rocha. 2011. Acute toxicity tests with the tropical cladoceran Pseudosida ramosa: the importance of using native species as test organisms. *Archives of Environmental Contamination and Toxicology* 60 (2):241-249.

Gardner, K. M. and T. V. Royer. 2010. Effect of Road Salt Application on Seasonal Chloride Concentrations and Toxicity in South-Central Indiana Streams. Journal of environmental quality **39**.

Garibay, R. and S. Hall. 2004. Threshold Recommendations for the Protection of Aquatic Life in the Upper Santa Clara River. Brentwood, TN.

Hamilton, Robert W, Joseph K Buttner, and Robert G Brunetti. 1975. Lethal levels of sodium chloride and potassium chloride for an oligochaete, a chironomid midge, and a caddisfly of Lake Michigan. *Environmental Entomology* 4 (6):1003-1006.

Harless, M. L., C. J. Huckins, J. B. Grant, and T. G. Pypker. 2011. Effects of six chemical deicers on larval wood frogs (*Rana sylvatica*). Environmental Toxicology and Chemistry **30**.

Harmon, S. M., W. L. Specht, and G. T. Chandler. 2003. A comparison of the daphnids *Ceriodaphnia dubia* and *Daphnia ambigua* for their utilization in routine toxicity testing in the Southeastern United States. Archives of Environmental Contamination and Toxicology **45**:79-85.

Hinton, M. J. and A. G. Eversole. 1979. Toxicity of ten chemicals commonly used in aquaculture to the black eel stage of the American eel. Pages 554-560 *in* Proceedings of the World Mariculture Society. Wiley Online Library.

Hoke, R. A., W. R. Gala, J. B. Drake, J. P. Giesy, and S. Flegler. 1992. Bicarbonate as a potential confounding factor in cladoceran toxicity assessments of pore water from contaminated sediments. Canadian Journal of Fisheries and Aquatic Sciences **49**:1633-1640.

Jackman, P. 2010. Email to M. Nowierski December 9. Reference toxicant data for NaCl tested on various aquatic organisms. Moncton Aquatic Toxicity Laboratory, Environment Canada. (Cited in Canadian Council of Ministers of the Environment 2011).

Khangarot, B. and P. Ray. 1989. Investigation of correlation between physicochemical properties of metals and their toxicity to the water flea *Daphnia magna* Straus. Ecotoxicology and Environmental Safety **18**:109-120.

King, K. and P. Farrell. 2002. Sensitivity of juvenile Atlantic sturgeon to three therapeutic chemicals used in aquaculture. North American Journal of Aquaculture **64**:60-65.

Komarudin, O., M. Shariff, and F. Shaharom. 1992. Toxicity of Sodium Chloride to *Clarias batrachus* (Linnaeus) and Its Effectiveness as a Chemotherapeutant for *Quadricanthus kobiensis* Ha Ky, 1968 Infections. Page 425 *in* Diseases in Asian aquaculture I: proceedings of the first Symposium on Diseases in Asian Aquaculture, 26-29 November 1990, Bali, Indonesia. Fish Health Section, Asian Fisheries Society.

Linton, T. K., C. Tarr, N. Rickramanayake, D. J. Soucek, and A. Dickinson. 2008. Acute Toxicity of Chloride to Select Freshwater Invertebrates. Great Lakes Environmental Center.

Loureiro, C., B. B. Castro, M. T. Claro, A. Alves, M. A. Pedrosa, and F. Goncalves. 2012. Genetic variability in the tolerance of natural populations of *Simocephalus vetulus* (Müller, 1776) to lethal levels of sodium chloride. Pages 95-103 *in* Annales de Limnologie-International Journal of Limnology. EDP Sciences.

Lowell, R. B., J. M. Culp, and F. J. Wrona. 1995. Toxicity testing with artificial streams: Effects of differences in current velocity. Environmental Toxicology and Chemistry **14**:1209-1217.

Martínez-Jerónimo, F. and L. Martínez-Jerónimo. 2007. Chronic effect of NaCl salinity on a freshwater strain of *Daphnia magna* Straus (Crustacea: Cladocera): a demographic study. Ecotoxicology and Environmental Safety **67**:411-416.

Meier, P. and B. Blastos. 2001. In: Evans. M. and Frick, C. 2001. The effects of road salts on aquatic ecosystems.

Meyer, J. S., D. A. Sanchez, H. L. Bergman, D. B. McWhorter, and J. A. Brookman. 1985. Chemistry and aquatic toxicity of raw oil shale leachates from Piceance Basin, Colorado. Environmental Toxicology and Chemistry **4**:559-572.

Mount, D. R. and D. D. Gulley. 1992. Development of a Salinity/toxicity Relationship to Predict Acute Toxicity of Saline Waters to Freshwater Organisms: Final Report. Gas Research Institute.

Mount, D. R., D. D. Gulley, J. R. Hockett, T. D. Garrison, and J. M. Evans. 1997. Statistical models to predict the toxicity of major ions to *Ceriodaphnia dubia*, *Daphnia magna* and *Pimephales promelas* (fathead minnows). Environmental Toxicology and Chemistry **16**:2009-2019.

Padhye, A. D. and H. V. Ghate. 1992. Sodium-chloride and potassium-chloride tolerance of different stages of the frog, *Microhyla ornata*. Herpetological Journal **2**:18-23.

Palmer, C., W. Muller, A. Gordon, P. Scherman, H. Davies-Coleman, L. Pakhomova, and E. De Kock. 2004. The development of a toxicity database using freshwater macroinvertebrates, and its application to the protection of South African water resources. South African Journal of Science **100**:643-650.

Pandolfo, T. J., W. G. Cope, G. B. Young, J. W. Jones, D. Hua, and S. F. Lingenfelser. 2012. Acute effects of road salts and associated cyanide compounds on the early life stages of the unionid mussel Villosa iris. Environmental Toxicology and Chemistry **31**.

Patrick, R., A. Scheier, and J. Cairns Jr. 1968. The relative sensitivity of diatoms, snails, and fish to twenty common constituents of industrial wastes. The Progressive Fish-Culturist **30**:137-140.

Sanzo, D. and S. J. Hecnar. 2006. Effects of road de-icing salt (NaCl) on larval wood frogs (*Rana sylvatica*). Environmental Pollution **140**.

Soucek, D. J., T. K. Linton, C. D. Tarr, A. Dickinson, N. Wickramanayake, C. G. Delos, and L. A. Cruz. 2011. Influence of water hardness and sulfate on the acute toxicity of chloride to sensitive freshwater invertebrates. Environmental Toxicology and Chemistry **30**:930-938.

Spehar, R. L. 1987. Memorandum to C. Stephan. U.S. EPA, Duluth, MN. June 24. U.S. EPA, Duluth, MN.

Thornton, Kent W. and John R. Sauer. 1972. Physiological Effects of NaCl on Chironomus attenuatus (Diptera: Chironomidae). *Annals of the Entomological Society of America* 65 (4):872-875.

Threader, R. and A. Houston. 1983. Use of NaCl as a reference toxicant for goldfish, Carassius auratus. Canadian Journal of Fisheries and Aquatic Sciences **40**:89-92.

Trama, F. B. 1954. The acute toxicity of some common salts of sodium, potassium and calcium to the common bluegill (*Lepomis macrochirus* Rafinesque). Proceedings of the Academy of Natural Sciences of Philadelphia **106**:185-205.

US EPA. 2010. Final report on acute and chronic toxicity of nitrate, nitrite, boron, manganese, fluoride, chloride and sulfate to several aquatic animal species.*in* U. S. E. P. Agency, editor., Office of Science and Technology, Health and Ecological Criteria Division, Region 5 Water Division.

Velasco-Santamaría, Y. M. and P. E. Cruz-Casallas. 2008. Behavioural and gill histopathological effects of acute exposure to sodium chloride in moneda (*Metynnis orinocensis*). Environmental Toxicology and Pharmacology **25**:365-372.

Viertel, B. 1999. Salt tolerance of Rana temporaria: spawning site selection and survival during embryonic development (Amphibia, Anura). Amphibia-Reptilia **20**:161-171.

Wang, N. and C. J. Ingersoll. 2010. Reference toxicity test data for NaCl and various aquatic invertebrates. United States Geological Survey. *in* M. Nowierski, editor.

Warne, M. S. J. and A. Schifko. 1999. Toxicity of laundry detergent components to a freshwater cladoceran and their contribution to detergent toxicity. Ecotoxicology and Environmental Safety **44**:196-206.

**Table S3.**  Results from phylogenetic signal analyses and Mantel tests.

|  | **Pagel's Lamda** | | | **Blomberg's K** | | **Mantel test** | |
| --- | --- | --- | --- | --- | --- | --- | --- |
| **Tree** | *Lambda* | *Log likelihood* | *P* | *K* | *P* | *Z* | *P* |
| All species | 0.690 | -386.96 | < 0.001 | 0.230 | 0.001 | 3738863.00 | 0.001 |
| Macroinvertebrate | 0.495 | -265.04 | 0.189 | 0.141 | 0.146 | 950864.50 | 0.673 |
| Amphibian | 0.000 | -114.51 | 1.000 | 0.313 | 0.359 | 221271.70 | 0.241 |
| Fish | 0.000 | -99.39 | 1.000 | 0.396 | 0.274 | 198893.00 | 0.407 |
| Chordata | 0.631 | -218.67 | < 0.001 | 0.436 | 0.001 | 1468457.00 | 0.001 |
| Arthropoda | 0.385 | -164.16 | 0.206 | 0.217 | 0.326 | 413637.10 | 0.279 |
| Mollusca | 0.000 | -72.70 | 1.000 | 0.325 | 0.438 | 51470.25 | 0.847 |
